# Efficient In Vitro Regeneration System and Comparative Transcriptome Analysis Offer Insight into the Early Development Characteristics of Explants from Cotyledon with Partial Petiole in Small-Fruited Pepper (*Capsicum annuum*)

**DOI:** 10.3390/ijms25147547

**Published:** 2024-07-09

**Authors:** Xiaoqi Li, Naveed Mushtaq, Na Xing, Shuhua Wu, Jiancheng Liu, Zhiwei Wang

**Affiliations:** 1 Key Laboratory for Quality Regulation of Tropical Horticultural Crops of Hainan Province, School of Breeding and Multiplication (Sanya Institute of Breeding and Multiplication), Center of Nanfan and High-Efficiency Tropical Agriculture, Hainan University, Sanya 572025, China; 21220951310154@hainanu.edu.cn (X.L.); naveedmushtaq@hainanu.edu.cn (N.M.); xn98123456@163.com (N.X.); 21210902000015@hainanu.edu.cn (S.W.); liujiancheng@hainanu.edu.cn (J.L.); 2 Key Laboratory for Quality Regulation of Tropical Horticultural Crops of Hainan Province, School of Tropical Agriculture and Forestry, Hainan University, Haikou 570228, China

**Keywords:** small-fruited pepper (*Capsicum annuum*), in vitro regeneration, hormones, histological observation, transcriptome

## Abstract

In our research, we utilized six small-fruited pepper germplasms as materials, selected cotyledons with the petiole and hypocotyls as explants, and conducted in vitro regeneration studies. Our outcomes specify that the most suitable explant is cotyledon with the petiole, and the suitable genotype is HNUCA341. The optimal medium for inducing and elongating adventitious buds for this genotype is Murashige and Skoog medium (MS) + 9.12 μM Zeatin (ZT) + 0.57 μM 3-Indoleacetic acid (IAA), with a bud induction rate of 44.4%. The best rooting induction medium is MS + 1.14 μM IAA, with a rooting rate of 86.7%. Research on the addition of exogenous hormones has revealed that the induction speed of buds in small-fruited pepper (HNUCA341) in the combination of ZT and IAA hormones (abbreviated as ZI) is quicker, and the induction effect is better. The histological observations indicate that ZI treatment accelerates the initiation of explant division and differentiation, causing a shorter duration of vascular-bundle tissue production. The plant hormone signaling pathway was significantly enriched by Kyoto Encyclopedia of Genes and Genomes (KEGG) analysis, including *ARR9* (LOC107843874, LOC107843885), *ARR4* (LOC107848380, LOC107862455), *AHK4* (LOC107870540), *AHP1* (LOC107839518), *LAX2* (LOC107846008), *SAUR36* (LOC107852624), *IAA8* (LOC107841020), *IAA16* (LOC107839415), *PYL4* (LOC107843441), and *PYL6* (LOC107871127); these significantly enriched genes may be associated with in vitro regeneration. In addition, the carbon metabolism pathway and plant mitogen-activated protein kinase (MAPK) signaling pathway are also significantly enriched in KEGG. The results of the Gene Ontology (GO) analysis revealed that differentially expressed genes related to carbon metabolism and fixation, photosynthesis and MAPK signaling pathways were upregulated under ZI treatment. It was found that they might be associated with enhanced regeneration in vitro. Furthermore, we also screened out differentially expressed transcription factors, primarily from the MYB, bHLH, AP2/ERF, and NAC families. Overall, our work accumulated important data for the in-depth analysis of the molecular mechanism of in vitro regeneration of pepper, and provides valuable germplasm for establishing an efficient stable pepper genetic-transformation system based on tissue culture.

## 1. Introduction

Pepper (*Capsicum annuum* L.) is the most extensively cultivated variety in the chili genus, and it comprises annual or perennial plants native to the tropical regions of Central and South America. Small-fruited pepper plants typically reach a height of 50–70 cm with robust branching yield and being relatively stable, making them suitable for use as condiments or ornamental plants with a wide range of applications. The plant genetic transformation technology can introduce target genes to enhance pepper traits, optimize genes rapidly, and improve various characteristics of pepper plants, crucial for research and practical purpose. The critical step in enhancing pepper varieties through genetic engineering is developing an efficient pepper regeneration system. At the same time, an early transcriptome analysis of in vitro regeneration was conducted to provide a theoretical reference for understanding the molecular mechanism of capsicum’s in vitro regeneration.

The plant regeneration process in vitro was greatly affected by genotypes, and the differentiation rate varies significantly among different genotypes. Studies have shown that genotypes induce significant differences in callus formation, adventitious buds, and adventitious roots [1]. A group of researchers compared the effects of cotyledon and hypocotyl as explants on the induction of adventitious buds in pepper plants. These studies discovered that the rate of bud induction was higher in cotyledon explants compared to hypocotyl explants [2]. Plant hormones play a crucial role in the in vitro regeneration process and regulate the growth and differentiation of plant cells, thereby influencing the entire regeneration process. In the in vitro culture of many plants, endogenous hormone levels are also considered a critical factor in bud formation and plant regeneration [3]. A better understanding of the relationship between the concentration of endogenous hormones in callus and the ability of adventitious bud formation in capsicum would benefit the in vitro reproduction of capsicum. Some other studies have shown that the imbalance between reactive oxygen species (ROS) production and cellular antioxidant capacity is one of the factors affecting plant regeneration ability [4,5,6]. At the same time, plant hormones can also regulate the synthesis of basic antioxidant enzymes, and the isoforms of some antioxidant enzymes are also related to the catabolism of plant hormones [7,8].

Several candidate genes related to in vitro regeneration have been identified in plants, and many researchers have discovered other genes that may be associated with in vitro regeneration. However, the specific functions of these genes are not yet clear enough. Further studies need to utilize genetic transformation technology to identify candidate genes to accurately comprehend these genes’ role in the process of in vitro regeneration. The identification and successful induction of regeneration-related genes can significantly enhance the regeneration rate and conversion rate of plants, which holds crucial practical application value.

This study established a small-fruited pepper in vitro regeneration system and regenerated plants in vitro. Two different concentrations of the exogenous hormone HNUCA341 were used for processing. The study compared the phenotype, histology, endogenous hormones, and enzyme activity changes. To identify the essential genes regulating in vitro regeneration of pepper cotyledon with petiole explants, we analyzed the transcriptomes treated with two different concentrations of the exogenous hormone HNUCA341. We found enrichment pathways related to in vitro regeneration, which establishes a foundation for a detailed analysis of the molecular mechanism of in vitro regeneration and the accumulation of data for the genetic transformation of pepper based on in vitro regeneration.

## 2. Results

### 2.1. Study on In Vitro Regeneration System of Capsicum

**Figure 1:**
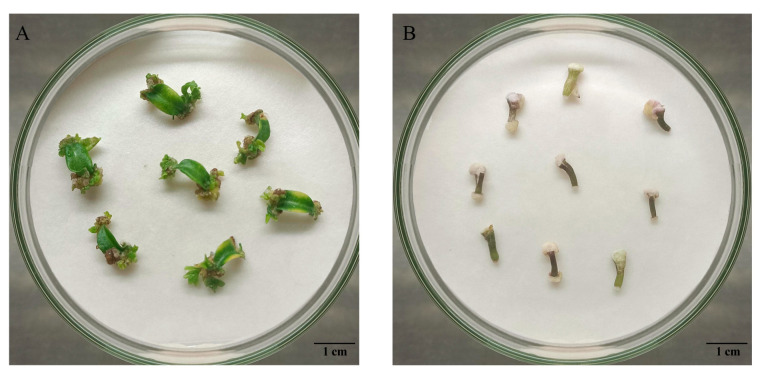
Bud induction manifestations in different explants. (**A**) Bud induction of cotyledon with petiole; (**B**) bud induction of hypocotyl.

The petiole of the small-fruited pepper undergoes dedifferentiation to form callus and generate adventitious buds at the base of the petiole under the influence of medium induction (Figure 1). In contrast, hypocotyl explants only enlarge to produce dense callus at the incision, which hinders the formation of adventitious buds. The results of the indeterminate bud differentiation rate indicate that leaf explants have rates above 20%, whereas hypocotyl explants have rates below 6% and are even unable to induce indeterminate buds at all (Table 1). The results showed that the adventitious bud induction rate was higher when using cotyledons as in vitro-regenerated explants than hypocotyls, and the induction effect was more pronounced (Figure 1).

**Figure 2:**
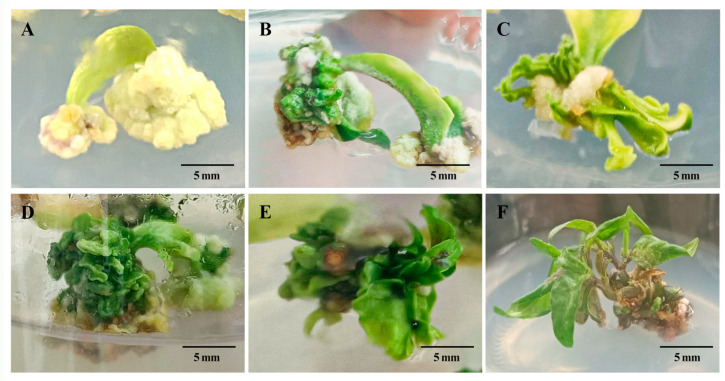
Induction of adventitious buds in culture medium. (**A**) Only white dense callus tissue is produced; (**B**–**D**) inducing the formation of deformed buds; (**E**) induced clustered buds; (**F**) normal adventitious buds.

The M9 medium exhibited the highest rate of bud induction among the four small-fruited peppers (HNUCA103, HNUCA146, HNUCA341, and HNUCA539) (Table 2). The buds produced continued to elongate. The induction effect was significantly superior to that of other media. The indefinite bud induction rate of HNUCA341 on this medium was 44.4%, considerably higher than that of the other medium (*p* < 0.05). The M6 medium had the most significant effect on the indefinite bud induction of HNUCA130 and HNUCA340, although the difference was not statistically significant compared to the M9 medium. The M1 induction medium had the lowest rate of bud induction. During the induction process, large number of the callus with yellow and white colors and a tight structure were mainly produced. It was difficult to achieve normal bud development, the most significant difference compared to the M9 induction medium. During the induction and elongation process of buds in small-fruited pepper, in addition to the normal buds, deformed buds were also produced at the petiole base. The leaves of buds failed to extend and unfold normally, and the clump buds could not continue to elongate (Figure 2).

**Figure 3:**
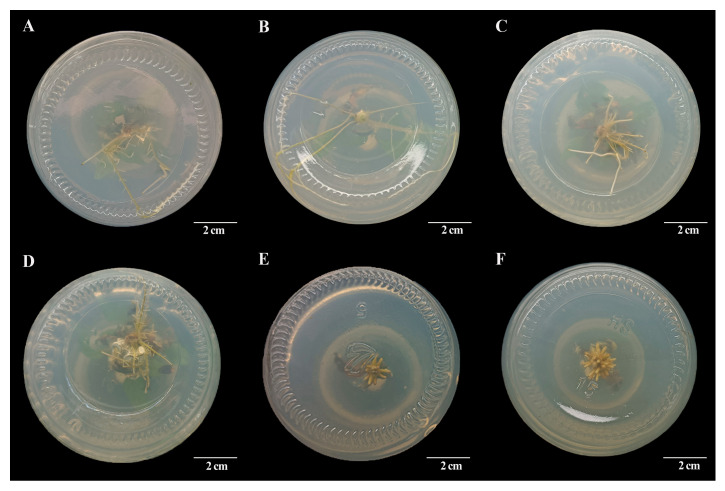
Rooting conditions of different rooting induction medium. (**A**) Rooting effect of R1 medium culture for 20 days; (**B**) rooting effect of R2 medium culture for 20 days; (**C**) rooting effect of R3 medium culture for 20 days; (**D**) rooting effect of R4 medium culture for 20 days; (**E**) rooting effect of R5 medium culture for 20 days; (**F**) rooting effect of R6 medium culture for 20 days.

The R2 medium exhibits the best rooting effect, with a rooting rate of up to 86%, resulting in slenderer roots and the good growth of subsequently regenerated plants (Table 3). The results of rooting induction showed that 1.14 μM of IAA was the optimal concentration to induce the rhizogenesis of indeterminate buds of chili pepper. The resulting roots were slender and abundant and could typically produce lateral roots and extend normally. The rooting effect exhibited a trend of initially increasing and then decreasing with the rise in the concentration of IAA. It is suggested that adding a high concentration of IAA to root culture is not conducive to the induction of adventitious buds. However, the root morphological characteristics induced by different concentrations of 1-Naphthylacetic acid (NAA) were mostly thicker and shorter, which was significantly different from normal roots (Figure 3). Moreover, with the increase in culture time, the roots were unable to continue expanding and did not produce lateral roots, resulting in a final rooting effect that was inferior to that of IAA.

### 2.2. Regenerated Pepper Seedling Transplanting

**Figure 4:**
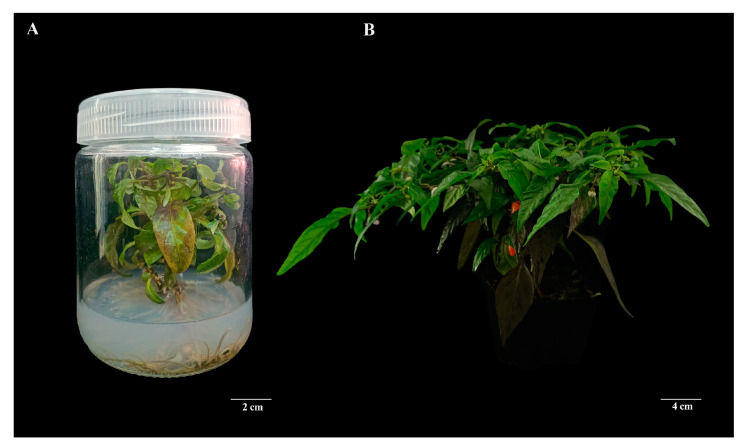
Regenerated pepper seedling transplanting. (**A**) Successfully regenerated pepper plants in the culturing flask; (**B**) regenerated plants after transplanting.

In the in vitro regeneration process, adventitious buds and adventitious roots are successfully induced from segments of pepper leaf explants to produce fully regenerated plants of small-fruited pepper. After the regenerated pepper plants grow to about 15 cm, they are transplanted into the culture medium and placed in an indoor incubator to adapt to the environment gradually. The indoor temperature of the culture room is maintained at around 25 °C, and the humidity is kept at about 70% to prevent the young leaves of the regenerated pepper seedlings from drying out and dying. After 1–2 weeks, the plants are moved from the outdoor environment once their growth stabilizes. The survival rate of regenerated pepper seedlings after transplanting was over 90%, and the regenerated seedlings were able to flower and bear fruit normally (Figure 4). This success indicates that the small-fruited pepper regenerated seedlings were cultivated successfully.

### 2.3. Analysis of Phenotype Characteristics of Explants at the Early Stage of In Vitro Regeneration

**Figure 5:**
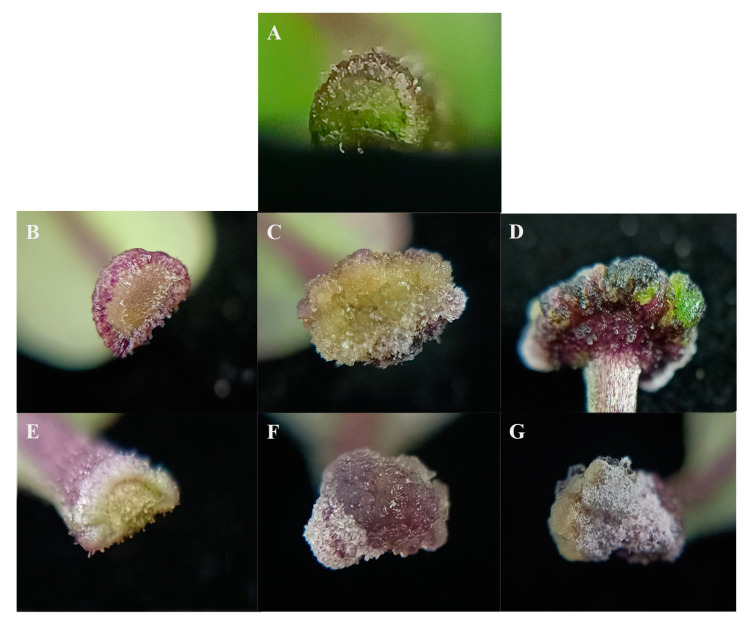
Early phenotypic characteristics of in vitro regeneration. (**A**) Untreated cotyledon with petiole base; (**B**) induction of ZI for 4 days; (**C**) induction of ZI for 8 days; (**D**) induction of ZI for 12 days; (**E**) induction of BI for 4 days; (**F**) induction of BI for 8 days; (**G**) induction of BI for 12 days.

According to the results of previous experiments, there were significant differences in the effect of bud induction under various hormone treatments through in vitro regeneration of small-fruited pepper. In this study, bud induction primarily occurred at the base of the petiole (Figure 5). Therefore, the two media groups with the most notable differences in bud induction effects were selected (M9 medium: 9.12 μM ZT + 0.57 μM IAA, ZI for short; M1 medium: 4.44 μM 6-Benzylaminopurine (6-BA) + 0.57 μM IAA, BI for short). HNUCA341, the most suitable genotype, was used as the material to compare the early phenotypes of handles of different hormone-induced regenerations in vitro. The phenotypes of indefinite bud induction at 0, 4, 8, and 12 days under various hormone treatments during the early stage of HNUCA341 were observed and analyzed. The outcomes showed that under ZI induction treatment, the petiole base of HNUCA341 was significantly enlarged, the cutting edge was swollen, and the entire stalk was noticeably thickened around the fourth day of the culture stage. The callus dedifferentiated at around eight days, and its size improved as the culture time prolonged. At around 12 days, bud points were generated at the edge of the petiole base, which could later differentiate into buds. Under BI induction treatment, the base of the petiole also enlarged at around four days, but it was significantly smaller than that under ZI treatment. The dedifferentiation of the left- and right-petiole bases on day eight resulted in callus formation, although the amount of callus was minimal. At around 12 days, only callus was present, with no bud points produced, and the changes in the callus were not obvious, and no significant increase was observed.

Under ZI treatment, it is evident that the speed of bud induction in small-fruited pepper is faster and the effect is more pronounced. At 12 days, the ZI treatment showed differentiated bud points, while the BI treatment only exhibited callus without any bud points.

### 2.4. Early Histological Features of In Vitro Regeneration

**Figure 6:**
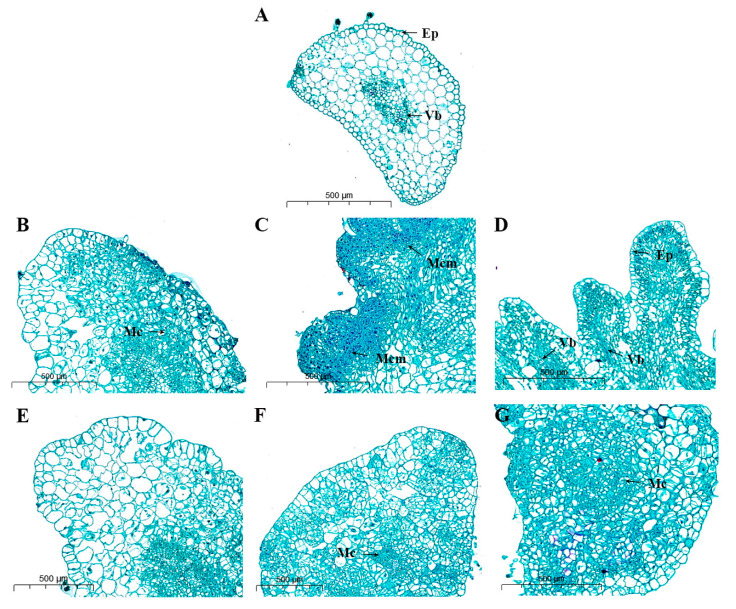
Early histological features of in vitro regeneration. (**A**) At the incision of the petiole of cotyledons that have not been cultured in vitro; (**B**) at the petiole incision of ZI cultured in vitro for 4 days, the upper epidermis and inner thin-walled cells undergo dedifferentiation; (**C**) inducing the formation of meristematic cell clusters near the incision of the petiole of ZI cultured in vitro for 8 days; (**D**) differentiation of meristematic cell clusters induced by 12 days of leaf stalk culture in ZI into bud primordia; (**E**) dedifferentiation of cells treated with BI for 4 days; (**F**,**G**) Continuous dedifferentiation of cells at 8d and 12d without obvious meristematic cell clusters. (Ep: epidermis; Vb: vascular bundle; Mc: meristem cells; Mcm: meristematic cell mass).

Under ZI treatment, cotyledons with the petiole explants of pepper began to divide, and the epidermis and parenchyma cells between the epidermis and vascular bundle dedifferentiated about four days after culture (Figure 6B). No evident epidermal cells have been invisible at around 8 days, and the cells dedifferentiated and divided into disordered and dense meristem cell clusters (Figure 6C). At around 12 days, the cells dedifferentiated, producing distinct vascular bundle tissues responsible for transporting water and nutrients (Figure 6D). Additionally, many bud primordia differentiated at the outer edge of the callus. After the BI induction treatment, the cell morphology changed around day four (Figure 6E). Although division was initiated, it was not as pronounced as the division induced by ZI, and the cells remained differentiated around days 8–12 (Figure 6F,G). Compared with the ZI-induced state, no bud primordia were generated, and no apparent meristem cell clusters were observed. The results showed that ZI treatment was more likely to induce the formation of meristem cell clusters and bud primordia under different hormone treatments of the same genotype. In contrast, BI treatment was more prone to continuous division, forming large calluses that have difficulty in forming buds. In this study, the histological observation of pepper organogenesis was conducted at the early stage of in vitro regeneration using different hormone combinations. A comparison of the differences and similarities in organogenesis under various hormone combinations was made to establish a histological basis for in vitro regeneration.

### 2.5. Changes in Endogenous Hormone and Enzyme Activity in Early Stage of In Vitro Regeneration

**Figure 7:**
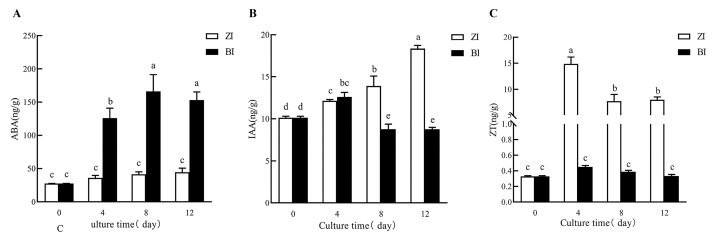
Comparison of endogenous hormone changes during in vitro regeneration process (**A**). Changes in endogenous hormones ABA levels; (**B**) changes in endogenous hormones IAA levels; (**C**) changes in endogenous hormones ZT levels. (Error bars denote SD. Different letters indicate significant differences among treatments at *p* < 0.05 level.).

**Figure 8:**
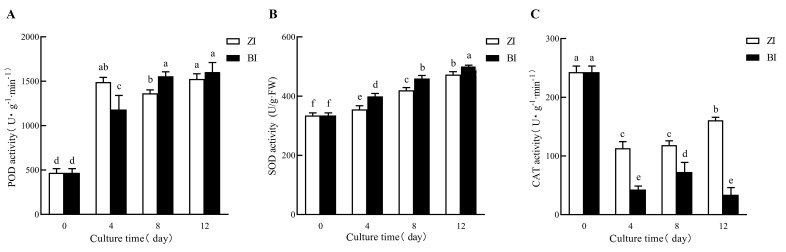
Comparison of enzyme activity changes during in vitro regeneration process. (**A**) Comparison of changes in POD enzyme activity; (**B**) comparison of changes in SOD enzyme activity; (**C**) comparison of changes in CAT enzyme activity. (Error bars denote SD. Different letters indicate significant differences among treatments at *p* < 0.05 level.).

The changes in endogenous hormone levels and enzyme activity are depicted in the figure. By comparing the changes in enzyme activity of HNUCA341 treated with two different hormone concentrations, it was observed that the endogenous Abscisic Acid (ABA) concentration under ZI treatment was lower than that under BI treatment at 4d, 8d, and 12d (Figure 7A, Supplementary Table S1). These outcomes propose that a lower concentration of endogenous ABA was more favorable for in vitro regeneration. Due to the addition of exogenous ZT, the concentration of endogenous ZT in the three periods was higher than that in the BI treatment, and ZI treatment had a more positive effect on adventitious bud induction, suggesting that a high concentration of endogenous ZT might be more promising for in vitro regeneration (Figure 7C). There was no significant difference in endogenous IAA concentration between ZI treatment for four days and BI treatment. However, the concentration was higher at 8–12 days compared to BI treatment (Figure 7B). The gradual increase in IAA concentration may be more conducive to in vitro regeneration. The effect of endogenous Gibberellin 3 (GA_3_) on plant regeneration in vitro may be minimal. Under ZI treatment, Superoxide dismutase (SOD) activity was lesser than under BI treatment at 4d, 8d, and 12d, though Catalase (CAT) activity was higher than under BI treatment (Figure 8B,C, Supplementary Table S2). These outcomes suggested that lower SOD activity and higher CAT activity may be more favorable for in vitro regeneration. Furthermore, the Peroxidase (POD) activity was higher in the ZI treatment for four days compared to the BI treatment but lower between eight and twelve days than the BI treatment (Figure 8A). Additionally, we have found that the POD with high activity in the early stage and low activity in the late stage may be more favorable for in vitro regeneration.

### 2.6. Identification of Differentially Expressed Genes

**Figure 9:**
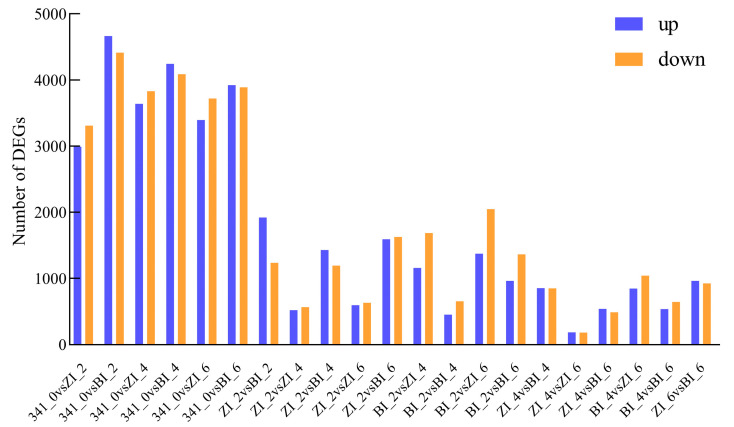
Number of differentially expressed genes.

**Figure 10:**
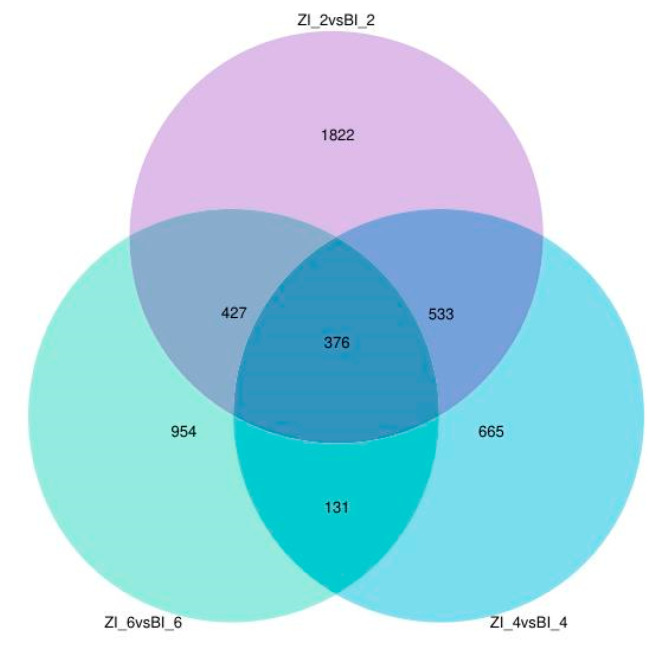
Differentially expressed genes of ZI_2vsBI_2, ZI_4vsBI_4 and ZI_6vsBI_6 in the Venn diagram.

The regulatory network of callus differentiation at the early stage of in vitro regeneration is a complex and delicate process involving the interaction of multiple biomolecules and signaling pathways. For a more profound comprehension, we selected the untreated stage and three developmental stages treated with different hormones (2d, 4d, 6d). Transcriptome sequencing was conducted on HNUCA341 explants the and induced callus of small-fruited pepper to screen for differentially expressed genes. Compared with the control group, a significant number of differentially expressed genes were identified in all three stages of regeneration following various hormone treatments. A total of 3158, 1705, and 1888 differentially expressed genes were identified in the ZI_2vsBI_2, ZI_4vsBI_4, and ZI_6vsBI_6 comparison groups (Figure 9). According to the Venn diagram, 376 differentially expressed genes were shared among the three groups. These 376 genes may promote regeneration in vitro (Figure 10).

### 2.7. Plant Hormone Signal Transduction Pathway

**Figure 11:**
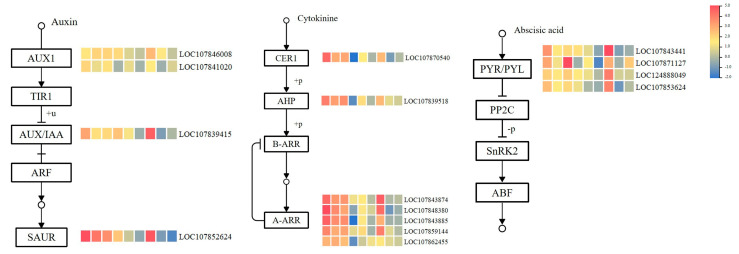
Candidate genes regulating in vitro regeneration of pepper in the hormone signal transduction pathway. The heat map from left to right represents ZI_2vsBI_2, ZI_4vsBI_4, ZI_6vsBI_6, 341_0vsZI_2, ZI_2vsZI_4, ZI_4vsZI_6, 341_0vsBI_2, BI_2vsBI_4, and BI_4vsBI_6.

According to the enrichment analysis of ZI_2vsBI_2, ZI_4vsBI_4, ZI_6vsBI_6, Kyoto Encyclopedia of Genes and Genomes (KEGG) metabolic pathways, and other studies, it was found that some significantly differentially expressed genes in plant hormone signaling pathways may be associated with the induction of plant regeneration in vitro. In the plant hormone signal transduction pathway, auxin, cytokinin, ethylene, and abscisic acid have received special attention, particularly in the cytokinin pathway (Figure 11). Significant differentially expressed genes were selected for heatmap visualization. These differentially expressed genes were all upregulated in response to ZI hormone treatment. The results showed that there are seven genes in the cytokinin signaling pathway: *ARR9* (LOC107843874, LOC107843885), *ARR4* (LOC107848380, LOC107862455), *ARR10* (LOC107859144), *AHP1* (LOC107839518), and *AHK4* (LOC107870540). Additionally, there are four genes in the auxin signal transduction pathway: *LAX2* (LOC107846008), *IAA8* (LOC107841020), *IAA16* (LOC107839415), and *SAUR36* (LOC107852624) and four genes in the abscisic acid signaling pathway: *PYL4* (LOC124888049, LOC107843441), *PYL6* (LOC107871127), and *PYL1* (LOC107853624).

### 2.8. Analysis of Early Transcription Factors in In Vitro Regeneration

Through the analysis of the early transcriptome of in vitro regeneration of small fruit capsicum, the discovery of transcription factors regulating in vitro regeneration of capsicum can provide a theoretical basis for developing an in vitro regeneration system for capsicum. In this study, a total of 505 differentially expressed transcription factors were found in ZI_2vsBI_2, ZI_4vsBI_4, and ZI_6vsBI_6, accounting for 7.5% of the total differentially expressed genes. These transcription factors belong to 55 transcription factor families, among which 290 genes were upregulated. In the three comparison combinations, there were 215 down-regulated genes, and the number of upregulated genes was higher than that of down-regulated genes. These transcription factor families mainly include MYB, bHLH, AP2/ERF, NAC, C2H2, HB, WRKY, etc. In the ZI_2vsBI_2 combinations, the number of upregulated and down-regulated genes accounted for approximately half of the total differentially expressed genes in the three comparison combinations.

### 2.9. GO and KEGG Enrichment

**Figure 12:**
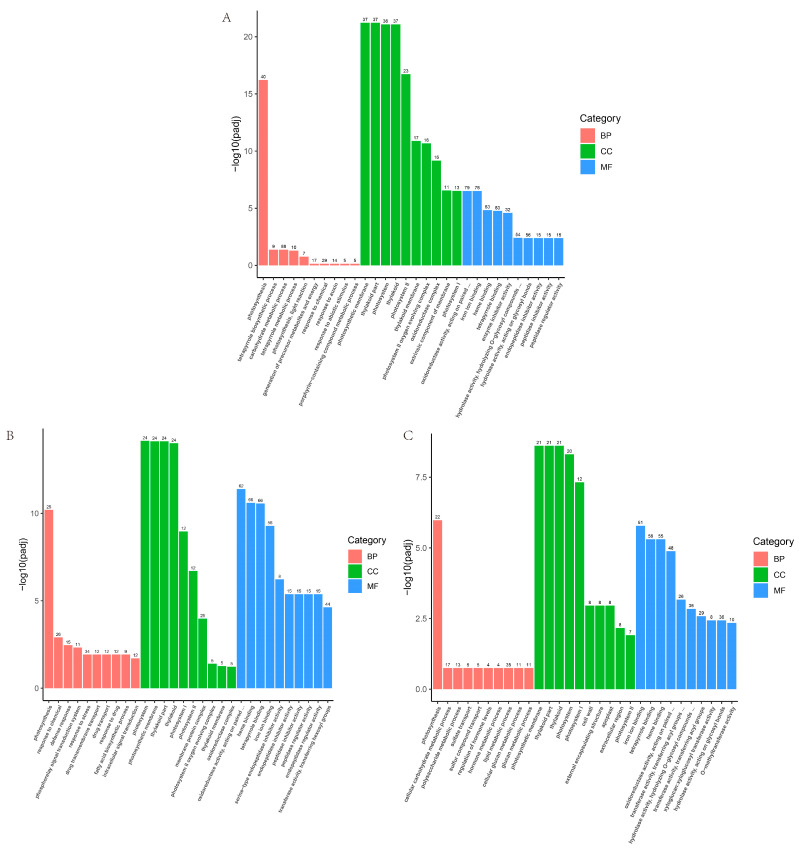
GO enrichment analysis of the differential genes. (**A**) GO enrichment of differential genes in ZI_2vsBI_2 group; (**B**) GO enrichment of differential genes in ZI_4vsBI_4 group; (**C**) GO enrichment of differential genes in ZI_6vsBI_6 group.

According to the Gene Ontology (GO) enrichment results, the similarity of GO enrichment outcomes in the three distinct comparative combinations was high, and it infers that in vitro regeneration might be associated with cell composition, such as photosystems and thylakoids. The genes related to photosynthesis, Oxidoreductase activity, and tetrapyrrole binding, and other functions may also play a role in plant regeneration in vitro (Figure 12).

**Figure 13:**
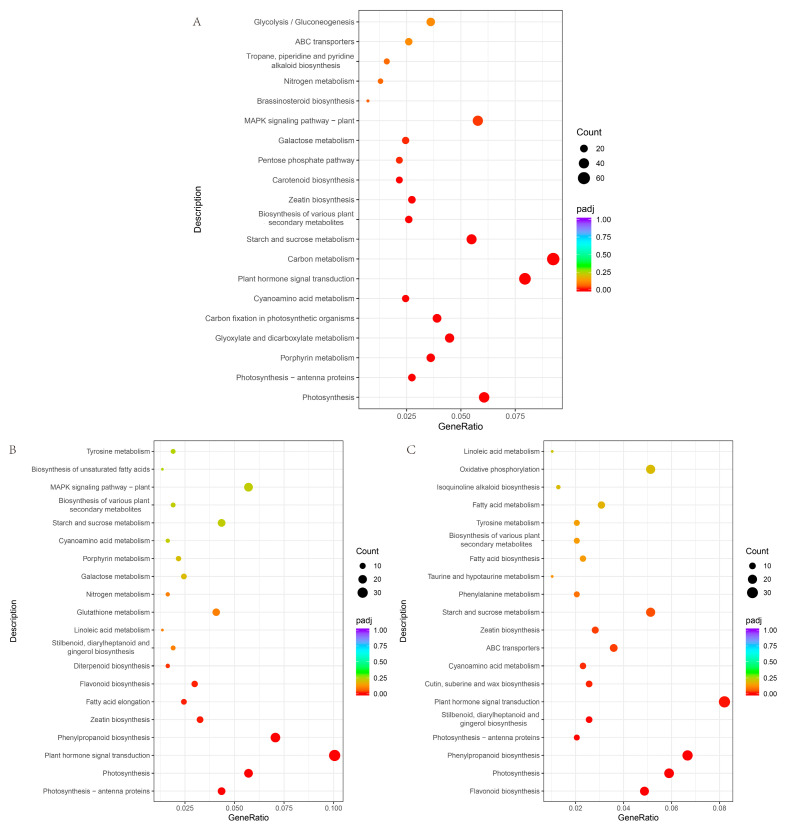
KEGG enrichment analysis of the differential genes. (**A**) KEGG enrichment of differential genes in ZI_2vsBI_2 group; (**B**) KEGG enrichment of differential genes in ZI_4vsBI_4 group; (**C**) KEGG enrichment of differential genes in ZI_6vsBI_6 group.

A total of 376 differentially expressed genes were identified in the ZI_2vsBI_2, ZI_4vsBI_4, and ZI_6vsBI_6 comparisons, distributed across 59 KEGG pathways (Figure 13). The most prevalent pathways included plant hormone signal-transduction and photosynthesis pathways. The three comparison combinations are highly enriched in the plant hormone signal transduction pathway, and the most differentially expressed genes are distributed in this pathway. These results suggest that the genes in the plant hormone signal transduction pathway are likely to be associated with the regeneration of small-fruited pepper in vitro.

### 2.10. In Vitro Regenerated Weighted Co-Expression Network Analysis

**Figure 14:**
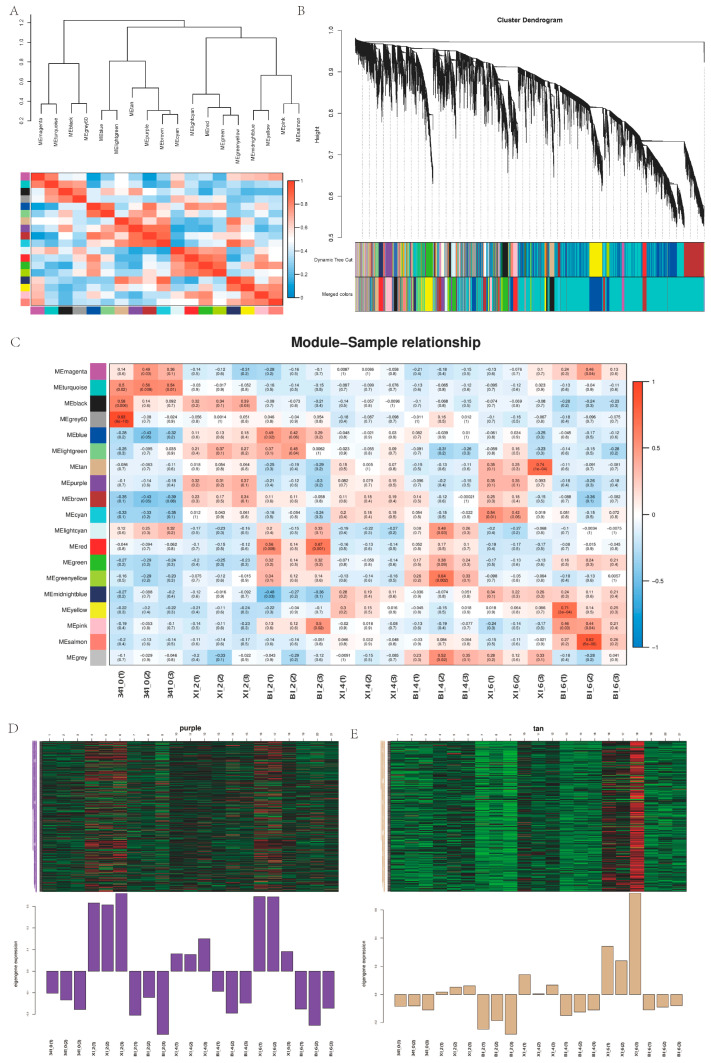
In vitro regenerated weighted co-expression network analysis. (**A**) Intermodular correlation heatmap; (**B**) hierarchical clustering of 19 modules with co expressed genes; (**C**) heat map of correlation between samples and modules; (**D**) gene expression level within the purple module; (**E**) gene expression level within the tan module.

**Figure 15:**
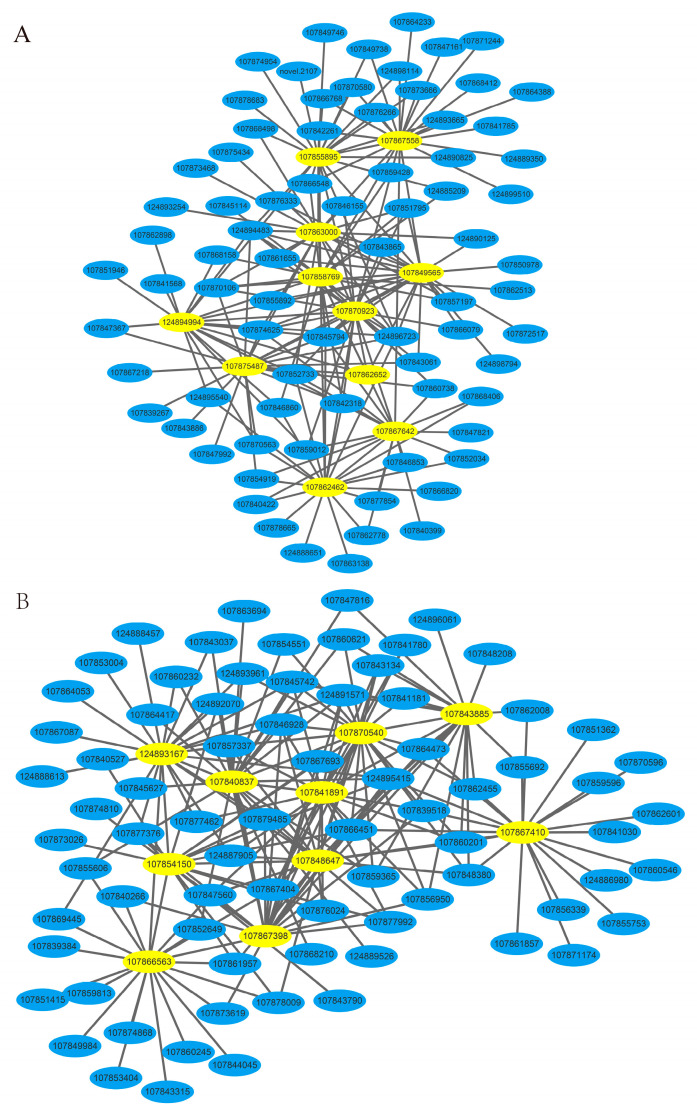
Gene-related network. (**A**) Tan module gene-related network; (**B**) purple module gene-related network.

Based on the RNA-seq data of three developmental stages and untreated stages of HNUCA341 in vitro regeneration, the transcription levels of buds induced by callus differentiation in the early stages of in vitro regeneration of small-fruited pepper were analyzed. The results showed that 22,926 differentially expressed genes were divided into 19 co-expression modules (Figure 14). The correlation analysis between modules and samples found that tan modules and purple modules are associated with in vitro regeneration. The two modules contained 291 and 360 differentially expressed genes, respectively, with most of the selected candidate genes being upregulated in the ZI hormone-treated state. The gene network association map was constructed for the core genes within the two modules (Figure 15, Supplementary Tables S3 and S4). It was found that 10 core genes were screened in the tan module. Noteworthy genes include LOC107870923, LOC107867642, LOC107849565, LOC107863000, LOC107855895, LOC107875487, LOC107867558, LOC107862462, LOC107858769, and LOC124894994. There are 10 core genes in the purple module to construct related networks for analysis. LOC107870540, LOC107843885, LOC107843885, LOC107867558, LOC107862462, LOC107858769, and LOC124894994. LOC107867398, LOC107841891, LOC107867410, LOC107840837, LOC107866563, LOC124893167, LOC107848647, and LOC107854150 are highly connected central genes. They may promote the regeneration of pepper explants in vitro.

### 2.11. Quantitative Real-Time PCR Validation

**Figure 16:**
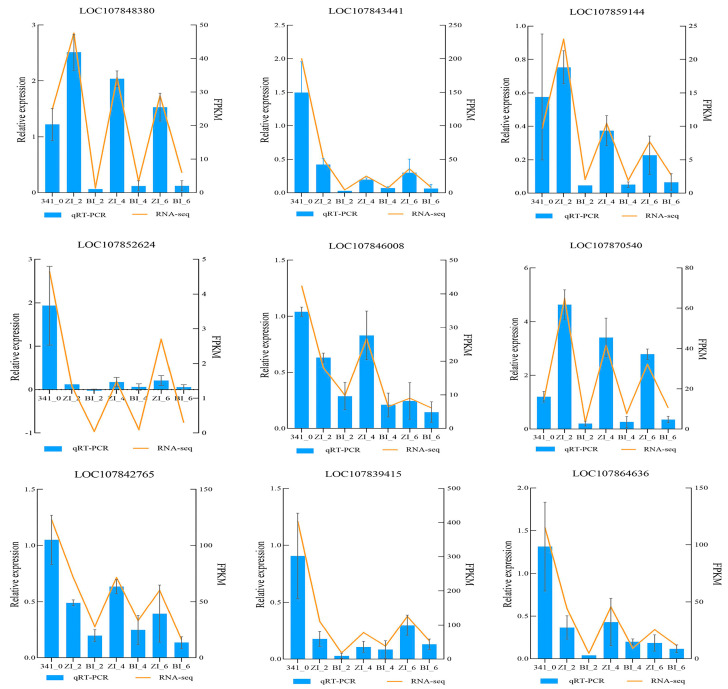
Results of qRT-PCR and RNA seq validation of differentially expressed genes in untreated stage and ZI, BI treatment for 2, 4 and 6 days. Error bars indicate SD.

To validate the reliability of the transcriptome data, nine differentially expressed genes closely associated with in vitro regeneration were selected based on the transcriptome sequencing data for qRT-PCR analysis. These genes include *ARR4* (LOC107848380), *PYL4* (LOC107843441), *ARR10* (LOC107859144), and others. The results showed that the changes in qRT-PCR results were almost identical to those in transcriptome sequencing data, representing a high reliability of the transcriptome data (Figure 16).

## 3. Discussion

The research on in vitro regeneration of pepper is relatively limited, and a comprehensive understanding of the gene regulation mechanism of pepper is crucial to establish a molecular basis for its genetic transformation. In this study, the potential for in vitro regeneration of six small-fruited pepper germplasms was investigated, and the medium suitable for in vitro regeneration of small-fruited pepper was screened. This discovery not only laid a foundation for genetic improvement and molecular-assisted breeding of small-fruited pepper, but also provided a new idea for exploring the molecular mechanism of in vitro regeneration of pepper. In addition, we analyzed the phenotype, histological characteristics, physiological indicators, and transcriptome of the regeneration induced by different hormone levels in small-fruited pepper, aiming to reveal the molecular regulatory mechanism of the process of bud formation in pepper and to further enhance our understanding in this field.

### 3.1. Construction of In Vitro Regeneration System of Pepper

Different genotypes exhibit significant variations in in vitro plant regeneration, making their genotypes one of the key factors influencing in vitro regeneration. Shafiq studied the response of four different pepper genotypes to in vitro regeneration and found that the rate of bud induction varied among the genotypes [9]. Orlinska studied the regeneration of various genotypes of four types of pepper and found that the regeneration rates varied [10]. Szasz studied the regenerative ability of 17 sweet pepper genotypes and found that the induced differentiation rate of adventitious buds significantly varied among different pepper genotypes [11].The addition of exogenous plant hormones and their concentration ratios is also one of the important factors affecting in vitro regeneration of pepper [12]. Therefore, selecting the appropriate exogenous hormones and concentration ratios is crucial to improving the induction rate of bud regeneration of small-fruited pepper in vitro. Previous studies have explored the induction of hormones for in vitro plant regeneration, with some researchers suggesting that the combination of Thidiazuron (TDZ) and IAA has the most effective impact on bud induction [13]. Kim found that the combination of 6-BA and NAA had the best induction effect [14]. The most commonly used combination is the hormone combination of 6-BA and IAA. During the process of plant regeneration in vitro, the addition of auxin can effectively induce the formation of plant roots. Moreover, increasing the auxin content appropriately can enhance the growth of roots [15]. Studies have confirmed that IAA and NAA are closely related to the rooting and growth of plants during the breeding process of plant regeneration in vitro. Some studies suggest that the root-inducing effect of adding IBA is more stable than that of NAA [16].

These outcomes showed that cotyledons with the petiole were the most efficient in inducing bud regeneration in pepper. Through this method, complete regenerated plants were obtained, but it was not easy to form buds and regenerate seedlings in hypocotyls. In this research, all six genotypes were capable of inducing buds, and among these, HNUCA146 predominantly induced callus, while cotyledons with the petiole of HNUCA341 exhibited the highest rate of adventitious bud induction at 44.44%. These results suggest that HNUCA341 is the most suitable genotype for in vitro regeneration of small-fruited pepper. The optimal medium concentration for bud induction was MS + 9.12 μM ZT + 0.57 μM IAA, which outperformed the medium combination of 6-BA and IAA. When the concentration of IAA was 1.14 μM, it was the optimal concentration to induce the rooting of pepper buds, and the rooting rate was up to 86.67%. The resulting roots were slender and could typically produce a large number of side roots. The rooting effect exhibited a pattern of initially increasing and then decreasing with the rise in the concentration of IAA. These outcomes suggest that excessively high concentrations of IAA in the rooting culture are not beneficial for the bud’s rooting process and these results laid the foundation for genetic transformation and molecular-assisted breeding of horticulture plants, especially the pepper plants. However, the varieties suitable for this in vitro regeneration system are not widely available, and hormone selection is a common exogenous hormone for in vitro regeneration. The influence of genotypes on in vitro regeneration remains significant. In the future, we can explore the use of a wider range of hormones, establish more detailed concentration gradients, and develop an in vitro regeneration system suitable for various pepper materials.

### 3.2. Early Histological Features of Explant Regeneration

In order to explore the characteristics of pepper in vitro regeneration, cotyledon with petiole in pepper were used as explants to observe the induction and development of cotyledon with petiole in vitro regeneration and to analyze the early process of in vitro regeneration from a microscopic point of view. A previous study reported cell division in the epidermal layer on the paraxial side of the explant center, near the most recent cut edge [17]. Histological analysis showed that cell division was observed in the epidermal and subepidermal layers [18]. Mezghani’s histological study provided evidence for the direct formation of organogenetic structures by epidermal cells in the petiolar incision of the cotyledon [19]. Ayzenshtat also provided a basis for the development of edge processes and the eventual formation of adventitious buds [20]. The results revealed that ZI treatment was more likely to induce the generation of meristem cell clusters and bud primordia in the epidermis and the parenchyma side between the vascular bundle and the epidermis. In contrast, BI treatment was more likely to continuously divide and form larger calluses, making it difficult to form buds. In this study, organogenetic processes of pepper were histologically observed under different exogenous hormones during the early stage of in vitro regeneration. The similarities and differences in the organogenetic processes of pepper were compared to establish a foundation for the histological aspects of in vitro regeneration.

### 3.3. Changes in Hormone and Enzyme Activity In Vivo at Early Stage of In Vitro Regeneration

The study on the effects of different endogenous hormones on the differentiation of explants during the induction differentiation stage of in vitro regeneration can reveal the key hormonal factors affecting the in vitro regeneration activity of small-fruited pepper. Previous study have shown that the induction rate of adventitious buds is consistent with the endogenous IAA hormone level [21]. Due to the heightened response of endogenous ABA to various stress treatments, it is believed to play a role in plant regeneration under stress conditions [22,23]. Some studies have shown that the accumulation of ABA inhibits the regeneration of immature barley embryo callus [24]. This is consistent with our results, indicating that low concentrations of endogenous ABA are more effective in in vitro regeneration. Wang et al. found that both endogenous IAA and zeatin gradually increased during regeneration [25]. By comparing the concentration changes of two different exogenous hormones HNUCA341, it was found that ZI treatment with a better in vitro regeneration effect detected a low concentration of endogenous ABA, a high concentration of endogenous ZT, and a gradually increasing concentration of IAA. This suggests that a low concentration of endogenous ABA, a high concentration of endogenous ZT, and a gradually increasing concentration of IAA may be more conducive to in vitro regeneration. The impact of endogenous GA_3_ on plant regeneration in vitro may be minimal. In summary, the in vitro regeneration process depends on the synergistic action of multiple hormones.

Plant cell differentiation typically involves various changes in metabolites and some researchers believe that the imbalance between the production of ROS and the antioxidant capacity of cells is one of the factors that affect the regenerative ability of plants [26]. In the process of callus growth induced by explants, the activity levels of three enzymes can serve as indicators of their proliferation [27]. In this study, the variations in enzyme activity during in vitro regeneration of pepper treated with different exogenous hormones were analyzed to explore the effect of enzyme activity on the in vitro regeneration of small-fruited pepper. Previous studies indicated that the increase in bud induction rate was accompanied by the increase in SOD, POD and CAT activities [28]. The results of endogenous hormone changes indicated that lower SOD activity, higher CAT activity and higher POD activity at the early stage, and lower activity at the later stage, may be more conducive to in vitro regeneration. It can be perceived that the activities of the three antioxidant enzymes fluctuated in coordination with each other during in vitro regeneration. Studying the changes in antioxidant enzyme activities during the in vitro regeneration of small-fruited pepper helps us to understand the internal vitality of cell division and differentiation.

### 3.4. Effect of Exogenous Hormone on Transcriptome Enrichment Pathway

Previous GO and KEGG analyses showed that differentially expressed genes associated with plant hormone signaling pathways, DNA replication, and amino and nucleotide sugar metabolism were significantly enriched at four stages of development during in vitro regeneration [29]. A previous study found that in the regeneration process of pepper, differential genes were primarily enriched in primary metabolism, followed by carbon metabolism and fixation, photosynthesis and amino acid metabolism, MAPK signaling pathway, hormone signal transduction, and other related pathways. The transcriptome results of this experiment also revealed that the differential genes in carbon metabolism and fixation, photosynthesis, MAPK signaling pathway, and hormone signal transduction-related pathways were upregulated under ZI treatment; consequently, the differential genes in these pathways could be associated with the in vitro regeneration of pepper. It found that the differentially expressed genes were mainly concentrated in the phenylpropane biosynthesis pathway and plant hormone signal transduction pathway in the KEGG enrichment analysis [30,31]. This is consistent with the results of KEGG enrichment in this experiment. KEGG enrichment analysis shows that plant hormone signal transduction plays a significant role in regulating pepper regeneration. A previous study revealed that auxin induces the transcription of multiple genes in Arabidopsis callus through a pathway that is initially involved in the expression of *ARF7* and *ARF19*. These genes play a crucial role in *Arabidopsis*’ dedifferentiation and callus formation [32]. The three comparison combinations were significantly enriched in the plant hormone signal transduction pathway, and the most differentially expressed genes were distributed in this pathway. This suggests that the genes in this pathway are likely associated with the in vitro regeneration of small-fruited pepper. In ZI and BI treatments, numerous differentially expressed genes were associated with plant hormone signal transduction. The genes involved in hormone signal transduction were primarily linked to auxin, cytokinin, ethylene, and abscisic acid, suggesting that plant hormones play a crucial role in callus regeneration. Consequently, there is potential for further investigation into the impact of key differentially expressed genes in these hormone pathways on the regeneration process in vitro.

In the early transcriptomic analysis results, the expression levels of auxin and abscisic acid candidate genes in the plant hormone signal transduction pathway showed that ZI treatment group was higher than BI treatment group at all time points. This finding was consistent with the results of plant hormone determination, further validating the accuracy of the experimental results.

### 3.5. Effects of Transcription Factors on Regeneration

In the process of in vitro plant regeneration, researchers have discovered some critical transcription factors that regulate regeneration. Previous studies have indicated that MYB transcription factor can induce the regeneration efficiency of Lagerstroemia indica callus [33]. MYB transcription factor is extensively involved in plant development and metabolism, controlling cell morphology [34]. In this study, the most significant number of MYB transcription factors were upregulated in all three comparison combinations, suggesting that MYB-TFs may be crucial for callus induction and regeneration. Due to the large number of MYB transcription-factor family members and their diverse roles in various plant species, further research is needed to understand the role of MYB in the in vitro regeneration of pepper. It was found that the heterologous expression of BBM in the AP2/ERF transcription factor family can improve the regenerative ability of tobacco [35]. Several studies have shown that *ESR1*, a member of the AP2/ERF gene family, is a crucial gene in the plant regeneration regulatory network of transcription factors, closely associated with cytokinin induction [36]. Moreover, AP2/ERF transcription factor WIND1 induces cell dedifferentiation and proliferation to form callus [37]. In the NAC family, *CUC1* and *CUC2* are regulated by several essential genes in the auxin and cytokinin-signal transduction pathways, which collectively enhance the plant’s ability for bud regeneration [38,39]. Overexpression of *CUC1* can significantly increase the regeneration rate of buds [40]. A previous study reported that *WRKY* is involved in callus formation [41]. Other studies have further shown that the transcription factor *WRKY* regulates callus development by regulating hydrogen peroxide content [42]. In this study, we found that several WRKY transcription factors were upregulated under ZI hormone treatment, indicating that WRKY plays an important role in regeneration. In addition, compared with other in vitro regeneration transcriptome results, we found that several bHLH transcription factors were upregulated under ZI hormone treatment, signifying that bHLH may also play a significant role in in vitro regeneration.

Compared with the results of Shu’s research, this research found that the hormone combination of ZT and IAA was more effective in inducing bud regeneration than 6-BA + IAA. Transcriptome analysis results indicate that the new bHLH, AP2/ERF, and NAC families may be associated with in vitro regeneration. Additionally, the results of GO enrichment were significantly different. However, the regeneration-related gene *WOX7*, identified by Shu, was not found in this study. Both studies suggested that 1.14 μM IAA was the most suitable for inducing rooting. Plant hormone signal transduction pathways are worthy of attention, and some of the differentially expressed genes may be related to in vitro regeneration [1].

## 4. Materials and Methods

### 4.1. Construction of In Vitro Regeneration System of Small-Fruited Pepper

Six small-fruited pepper germplasm HNUCA103, HNUCA130, HNUCA146, HNUCA340, HNUCA341, and HNUCA539 were selected to plant sterile seedlings. Cotyledons with petioles and hypocotyls of 0.5–1 cm were removed from well-grown sterile pepper seedlings and inoculated on culture medium with different hormone ratios for adventitious bud induction, with 30 explants per combination. All experiments were repeated three times, and the induction rate of adventitious buds was calculated 30 days later to select the most suitable explants for in vitro regeneration. The optimal cut explants were placed in an adventitious bud-induction medium with different concentration ratios, with 30 explants for each treatment concentration. After 30 days, the adventitious bud-induction rate of the explants was calculated using the concentration reference settings of İzgü and different concentration gradients (Table 4) to screen the most suitable hormone concentration for adventitious bud induction and elongation of small-fruited pepper [43]. Different concentrations of NAA and IAA plant hormones were selected to explore the optimal rooting medium for small-fruited pepper (Table 5) [1]. The results are indicated as mean ±SD of three duplications.

### 4.2. Phenotypic and Histological Analysis

Based on the previous experimental results, it was found that the small-fruited pepper HNUCA341 produced buds approximately 12 days after the hormone ratio of MS + 9.12 μM ZT + 0.57 μM IAA treatment. The effect of MS + 4.44 μM 6-BA + 0.57 μM IAA treatment differed the most from the optimal condition, resulting in the production of only large calluses. Therefore, the callus-induced early differentiation was selected by adding the above two hormone ratios at three different stages of 4, 8, and 12 days. The callus was collected in a 50% FAA solution. Histological observations were made with reference to previous sections and staining methods [44]. Paraffin sections were first dewaxed, followed by environmental dewaxing transparent liquid I for 20 min, environmental dewaxing transparent liquid II for 20 min, anhydrous ethanol I for 5 min, anhydrous ethanol II for 5 min and 75% alcohol for 5 min, and then rinsed with tap water. The slices were dipped into the plant saffranine dyeing solution for 2 h and the excess dye washed off with running water. Then the sample was immersed in 50%, 70%, and 80% gradient alcohol for 3–8 s each for decolorization. Subsequently, it was placed it in the plant solid green dyeing solution for 6–20 s, and in anhydrous ethanol for three-cylinder dehydration. Finally, it was immersed in clean xylene for 5 min for transparency, and sealed with neutral gum on a slide. Microscopy, image acquisition, and analysis then proceeded.

### 4.3. Measurement of Endogenous Hormone and Enzyme Activity

According to the regeneration results, the explants and callus underwent differentiation with 4.44 μM 6-BA + 0.57 μM IAA and 9.12 μM ZT + 0.57 μM IAA. Samples were collected at three stages (4d, 8d, and 12d) as experimental materials for the bud differentiation process, and the physiological indexes were determined and analyzed. Triplicate biological replicates were made for each sample. The enzyme activities of SOD, CAT, and POD, as well as the changes in hormones such as zeatin, gibberellin, indoleacetic acid, and abscisic acid during adventitious bud differentiation under various hormone treatments, were measured.

SOD, POD, and CAT activities were measured using the Superoxide Dismutase Activity Detection Kit (SE001-C48, Imagene, Beijing, China), the Peroxidase Activity Detection Kit (SE002-C48, Imagene, Beijing, China), and the Catalase Activity Detection Kit (UV method) (SE003-C48, Imagene, Beijing, China), all of which were obtained from Beijing Codon Biotechnology Co., Ltd. (CODONX, Beijing, China).

Plant hormones were isolated and analyzed using high-performance liquid chromatography. The concentration of endogenous auxin was determined according to the method described previously [45]. After the sample was ground into powder with liquid nitrogen, the 1.0 g sample was weighed into a 15 mL centrifuge tube. Then, 5 mL of extraction solution (80% methanol-0.5% formic acid aqueous solution) was added. The samples were soaked overnight at 4 °C and then extracted by ultrasonic shock. After centrifugation, the supernatant was obtained, and 3 mL of extraction liquid was added for repeated extraction. The supernatant was mixed, and then exactly 7 mL was removed and diluted 5 times with distilled water for purification. The MCX solid-phase extraction column was activated and balanced with 5 mL methanol and 5 mL water, respectively. The liquid was then passed through the column, washed with 6 mL of 2% formic acid water, eluted with 10 mL of 5% ammoniated methanol, and the eluent was collected. The eluent was dried with liquid nitrogen, redissolved with 1 mL of 20% methanol aqueous solution, and the supernatant was centrifuged into a brown sample bottle and then injected into an ultra-high-performance liquid chromatography–triple quadrupole tandem mass spectrometer (Thermo Altis Plus, Thermo Fisher Scientific, Waltham, MA, USA). The mixed standard solution was diluted step by step into a series of standard solutions, and the standard curve was drawn with the standard concentration x (ng/mL) as the horizontal axis and the reference peak area y as the vertical axis to determine the content of the target substance in the sample. All data were processed using TraceFinder 5.1 chemical workstations.

### 4.4. Transcriptome Sampling and Sequencing

The transcriptome analysis of callus induced by small-fruit pepper germplasm HNUCA341 at different early culture stages was studied under two distinct hormone treatments. The samples were collected from four groups of explants and callus under different hormone treatments at four stages: 0 days without treatment (named 341_0), 2 days of culture time (named ZI_2 for ZI treatment and BI_2 for BI treatment), 4 days of culture time (named ZI_4 and BI_4), and 6 days of culture time (named ZI_6 and BI_6). Three samples were collected for each group and the protocol for transcriptome analysis involved placing the explants in a medium with the appropriate hormone concentration ratio for induction culture. The culture conditions included 16 h of light and 8 h of darkness, with a temperature of 25 °C. Twenty-one libraries were prepared according to Illumina’s RNA kit and the FastQC employed for quality control of each library. All samples were tested, and the filtered clean read data were compared with the NCBI annual pepper reference genome.

### 4.5. Differential Gene Screening and Enrichment Analysis

The differentially expressed genes among several samples were calculated and analyzed using the DESeq2 R package (v.1.18.1) [46]. The criteria for screening differentially expressed genes were |log2(fold change)| ≥ 1, and a *p*-value ≤ 0.05 was considered significant for enrichment. The KEGG database was utilized to identify enrichment pathways of differentially expressed genes, and GO enrichment analysis was conducted to determine the primary pathways and functional categories of differentially expressed genes in various comparisons [47]. Through KEGG analysis results, key genes were selected from the pathways that were significantly enriched and focused on in the three analysis combinations, and the heatmap was visualized by the pheatmap package in the R language.

### 4.6. WGCNA Gene Co-Expression Network

Weighted correlation network analysis (WGCNA) was utilized to analyze the gene expression patterns in various samples using the R programming language. This method helped identify gene modules with similar expression patterns. In this study, the FPKM value in the samples was used to measure the soft threshold. Cytoscape (v3.10.1) was used to build the gene co-expression network, illustrating the gene correlations within each module for further analysis.

### 4.7. Quantitative Real-Time PCR Validation

To confirm the reliability of the transcriptome sequencing data, 9 differentially expressed genes closely related to in vitro regeneration were selected for qRT-PCR analysis. The RNA extraction kit was used to extract RNA from callus generated by HNUCA341 treated with different hormones. Furthermore, the quality of the RNA was verified, and then converted into the cDNA. In our study we used three replicate samples for each sample material. The primers used in this study were designed using the software Primer5.0 and validated with Oligo7 and Prime-BLAST (Supplementary Table S5). The primers were also further screened by agarose gel electrophoresis.

## 5. Conclusions

A comprehensive understanding of the genes associated with adventitious bud formation induced by pepper explants is essential for comprehending the regeneration process of pepper. This study successfully established an efficient in vitro regeneration system for small-fruited pepper. In addition, we performed histological, endogenous hormone, enzyme activity, and transcriptomic analysis of peppers in the in vitro regeneration process. These findings lay the foundation for an in-depth analysis of the molecular mechanism of capsicum regeneration and genetic transformation based on in vitro regeneration for future studies.

## Figures and Tables

**Table 1:** The induction rate of buds from different explants.

Name	The Induction Rate of Cotyledon with the Petiole (%)	The Induction Rate of Hypocotyl (%)
HNUCA103	28.89 ± 5.09 b	5.56 ± 1.93 a
HNUCA130	23.33 ± 3.34 b	1.11 ± 1.92 ab
HNUCA146	23.33 ± 3.34 b	0 c
HNUCA340	21.11 ± 5.09 b	3.33 ± 3.34 ab
HNUCA341	44.44 ± 1.93 a	0 c
HNUCA539	25.56 ± 5.09 b	5.55 ± 3.85 a

Note: The numerical value represents the mean ± SD value; the same letter indicates no significant difference in Duncan analysis (*p* < 0.05). Lowercase letters indicate that the difference is not significant if there is one identical marker letter and the difference is significant if there is a different marker letter.

**Table 2:** Effects of different induction media on the induction of buds in pepper.

No.	HNUCA103	HNUCA130	HNUCA146	HNUCA340	HNUCA341	HNUCA539
M1	4.45 ± 3.85 e	2.22 ± 1.92 e	1.11 ± 1.92 f	1.11 ± 1.92 f	0 g	0 f
M2	7.78 ± 1.92 de	10.00 ± 3.33 bcde	8.89 ± 6.94 de	6.67 ± 3.34 ef	0 g	0 f
M3	11.11 ± 1.92 d	10.00 ± 3.33 bcde	13.33 ± 3.34 bcd	15.56 ± 1.93 abcd	17.78 ± 1.92 e	17.78 ± 5.09 bcde
M4	12.22 ± 5.09 d	11.11 ± 6.94 bcd	6.67 ± 3.34 de	16.67 ± 3.34 abcd	21.11 ± 1.92 de	18.89 ± 1.92 bcd
M5	14.44 ± 1.93 cd	12.22 ± 3.85 bcd	11.11 ± 6.94 cd	18.89 ± 5.09 abc	26.67 ± 3.34 c	16.67 ± 3.34 cde
M6	27.78 ± 1.92 a	23.33 ± 3.34 a	20.00 ± 3.33 ab	22.22 ± 5.09 a	34.44 ± 1.93 b	20.00 ± 3.33 abc
M7	13.33 ± 3.34 d	17.78 ± 5.09 ab	14.44 ± 5.09 bcd	11.11 ± 5.09 de	26.67 ± 3.34 c	13.33 ± 3.34 de
M8	20.00 ± 3.33 bc	21.11 ± 5.09 a	21.11 ± 5.09 ab	20.00 ± 3.33 ab	25.56 ± 5.09 cd	25.56 ± 5.09 a
M9	28.89 ± 5.09 a	16.67 ± 3.34 abc	23.33 ± 3.34 a	21.11 ± 5.09 ab	44.44 ± 1.93 a	20.00 ± 3.33 abc
M10	21.11 ± 1.92 b	15.56 ± 1.93 abc	18.89 ± 1.92 abc	17.78 ± 1.92 abcd	34.44 ± 1.93 b	23.33 ± 3.34 ab
M11	12.22 ± 1.92 d	8.89 ± 5.09 cde	17.78 ± 5.09 abc	14.44 ± 1.93 bcd	20.00 ± 3.33 e	12.22 ± 1.92 e
M12	10.00 ± 6.67 de	6.67 ± 3.34 de	14.44 ± 5.09 bcd	12.22 ± 1.92 cde	12.22 ± 3.85 f	17.78 ± 1.92 bcde

Note: The numerical value represents the mean ± SD value; the same letter indicates no significant difference in Duncan analysis (*p* < 0.05). Lowercase letters indicate that the difference is not significant if there is one identical marker letter and the difference is significant if there is a different marker letter.

**Table 3:** The effect of different culture media on the induction of pepper rooting.

No.	Rooting Rate
R1	22.22 ± 5.09 cd
R2	86.67 ± 3.33 a
R3	43.33 ± 3.33 b
R4	35.56 ± 3.85 b
R5	15.56 ± 5.09 d
R6	24.44 ± 5.09 c

Note: The numerical value represents the mean ± SD value; the same letter indicates no significant difference in Duncan analysis (*p* < 0.05). Lowercase letters indicate that the difference is not significant if there is one identical marker letter and the difference is significant if there is a different marker letter.

**Table 4:** Ratio of hormone concentration in pepper induction medium.

No.	Hormone Concentration (μM)		
	6-Benzylaminopurine (6-BA)	Zeatin (ZT)	3-Indoleacetic Acid (IAA)
M1	4.44	0	0.57
M2	4.44	0	2.85
M3	8.88	0	0.57
M4	8.88	0	2.85
M5	13.32	0	0.57
M6	13.32	0	2.85
M7	0	4.56	0.57
M8	0	4.56	2.85
M9	0	9.12	0.57
M10	0	9.12	2.85
M11	0	13.68	0.57
M12	0	13.68	2.85

Note: each treatment contained 30 explants and triplicate biological replicates for each sample.

**Table 5:** Formula of rooting induction medium for small-fruited pepper.

No.	Rooting Medium
R1	MS + 0.57 μM IAA
R2	MS + 1.14 μM IAA
R3	MS + 2.85 μM IAA
R4	MS + 0.54 μM NAA
R5	MS + 1.07 μM NAA
R6	MS + 2.69 μM NAA

Note: each treatment contained 30 explants and triplicate biological replicates for each sample.

## Data Availability

Data are contained within the article and Supplementary Materials.

## Supplementary Materials

The following supporting information can be downloaded at: https://www.mdpi.com/article/10.3390/ijms25147547/s1.
